# Supplementary material for: ViraLite: An Ultracompact HIV Viral Load Self-Testing System with Internal Quality Control
Source: medRxiv. 2025 Apr 3:2025.04.01.25325036. Preprint. [Version 1] doi: 10.1101/2025.04.01.25325036 (PMC11998842; doi:10.1101/2025.04.01.25325036)
Supplement: Supplementary file 1 [file media-1.pdf]

ViraLite: An Ultracompact HIV Viral Load Self-Testing System with Internal Quality Control

Anthony J. Politza[1], Tianyi Liu[2], Aneesh Kshirsagar[2], Ming Dong[2], Md. Ahasan Ahamed[2,6], Muhammad Asad Ullah Khalid[6], Roland Jones[3], Uttara Seshu[4], Kathryn Risher[4], Casey N. Pinto[4,5], Yusheng Zhu[3], and Weihua Guan[6*]

1. Department of Biomedical Engineering, Pennsylvania State University, University Park, PA, United States
2. Department of Electrical Engineering, Pennsylvania State University, University Park, PA, United States
3. Departments of Pathology and Laboratory Medicine and Pharmacology, Milton S. Hershey Medical Center and Pennsylvania State University College of Medicine, Hershey, PA, United States
4. Department of Public Health Sciences, Pennsylvania State University College of Medicine, Hershey, PA, United States
5. The Pennsylvania State University Cancer Institute, Cancer Control Program, Hershey, PA, United States
6. Department of Intelligent Systems Engineering, Indiana University, Bloomington, Indiana 47408

* Corresponding email address: guanw@iu.edu

Contents

Supplementary Information....................2

Supplementary Figures....................4

Supplementary Tables....................9

Supplementary Videos....................12

Supplementary Information

RT-LAMP Design. Earlier studies into multiplexed LAMP have demonstrated probe-based detection using labeled primers[1–3]. Detection of Amplification by Releasing of Quenching (DARQ) supplements the standard LAMP primers with a duplex probe[4,5]. In this method the duplex consists of a quencher attached forward inner primer (QFIP) annealed to a fluorophore labeled F1 region (Fd)[4]. Another method of probe-based LAMP, 5p-OSD, also supplements the standard LAMP assay with a duplex probe[6,7]. The difference between DARQ and 5p-OSD is apparent in the utilization of one of the standard loop primers (LP) as one half of the added duplex[7]. In this manner, 5p-OSD is designed with a 5'-fluorophore and annealed to a complementary strand containing a 3' quencher (Qd) . Our design uses both DARQ (HIV-1) and 5p-OSD (RNase P) to achieve a multiplex LAMP assay for HIV-1 and RNase-P.

Analyzer Design. In a previous study we showed that using single color channels to monitor bulk assay fluorescence limited the capabilities of multiplexing [8] (**Supplementary Figure S5**). However, using a multi-spectra sensor enables ViraLite to analyze broad spectrum output from an assay and then a trained machine learning model can assist in the conversion from 8 channel signal to 2 fluorophore concentrations. The handheld device is constructed using 3D-printed ABS to support the electrical and thermal modules. Inside the device, a 7.4V 2200mAh Li-ion battery provides power to the motherboard, resistive heater, and Arduino MCU. The MCU controls the excitation LEDs, indication LEDs, resistive heating module, and optical array. Excitation light from the blue LEDs (480 nm) enters the heating block from the bottom where the incident light hits the assay tube. Then, emitted light from the assay is detected by color sensors positioned 90° off axis of the excitation light.

Survey information. A significant majority of our survey participants were male (60%) with a minority population of female and other (transgender male, transgender female, non-binary, other). We found most of our participants were between 25-34 with an average income between $50,000-99,000. Our results showed that for the plurality of participants the highest education level among participants was a bachelor's degree. The most common environment for

respondents was an urban setting and our surveyed population was primarily white with small but present representation of African American and Hispanic populations.

Supplementary Figures

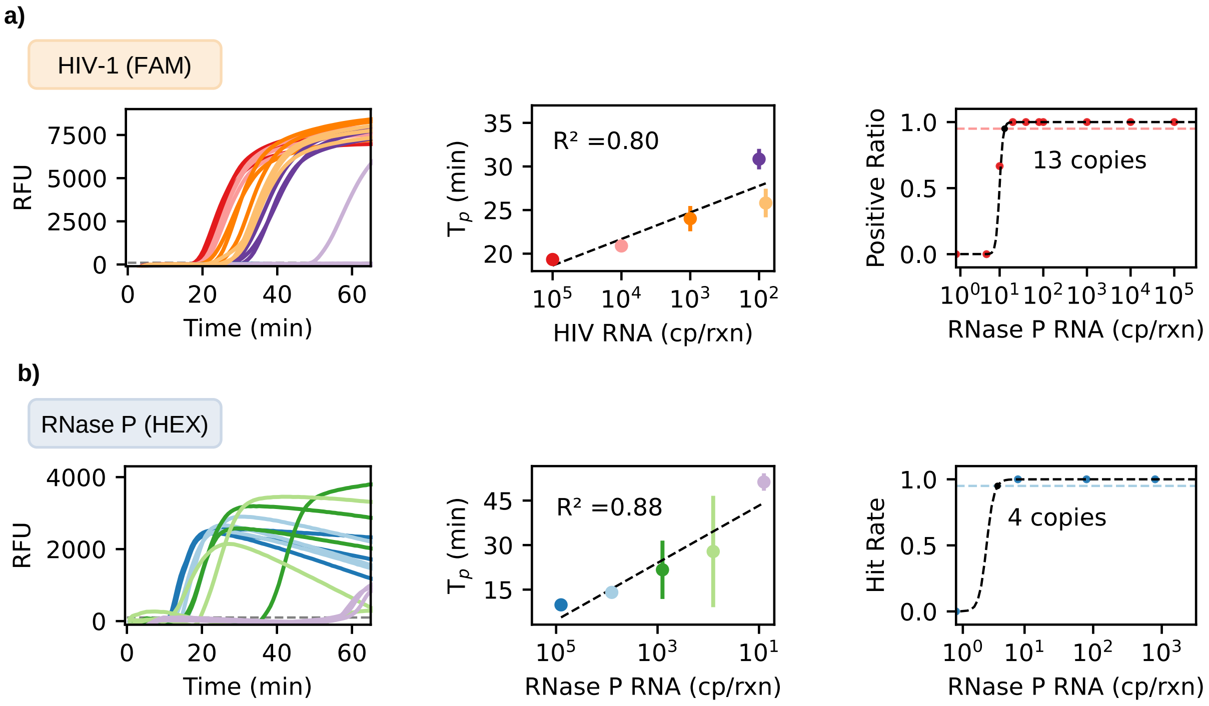

Figure S1. Single-plex assay validation. a) The probe-based HIV-1 Assay was initially screened using 10^5 down to 1 copy of viral RNA. The summary of detection linearity showed an R^2 of 0.80 and resulted in a limit of detection of 13 copies (95% CI). b) Similarly, the probe-based RNase P assay was screened using 10^5 down to 1 copy of viral RNA. The summary of detection linearity showed an R^2 of 0.88 and resulted in a limit of detection of 4 copies (95% CI).

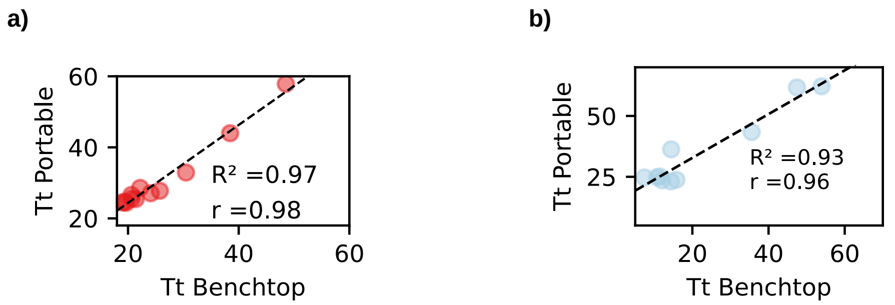

Figure S2. Single-plex correlation using portable vs benchtop. a) Our HIV-1 assay showed very strong correlation (r = 0.98) between analyzer formats and great linearity (R^2 = 0.97) for semi-quantitative analysis. b) Our RNase P assay also showed very strong correlation (r = 0.96) between analyzer formats and great linearity (R^2 = 0.93) showcasing the potential for multiplex detection between both assays, regardless of sensitivity losses.

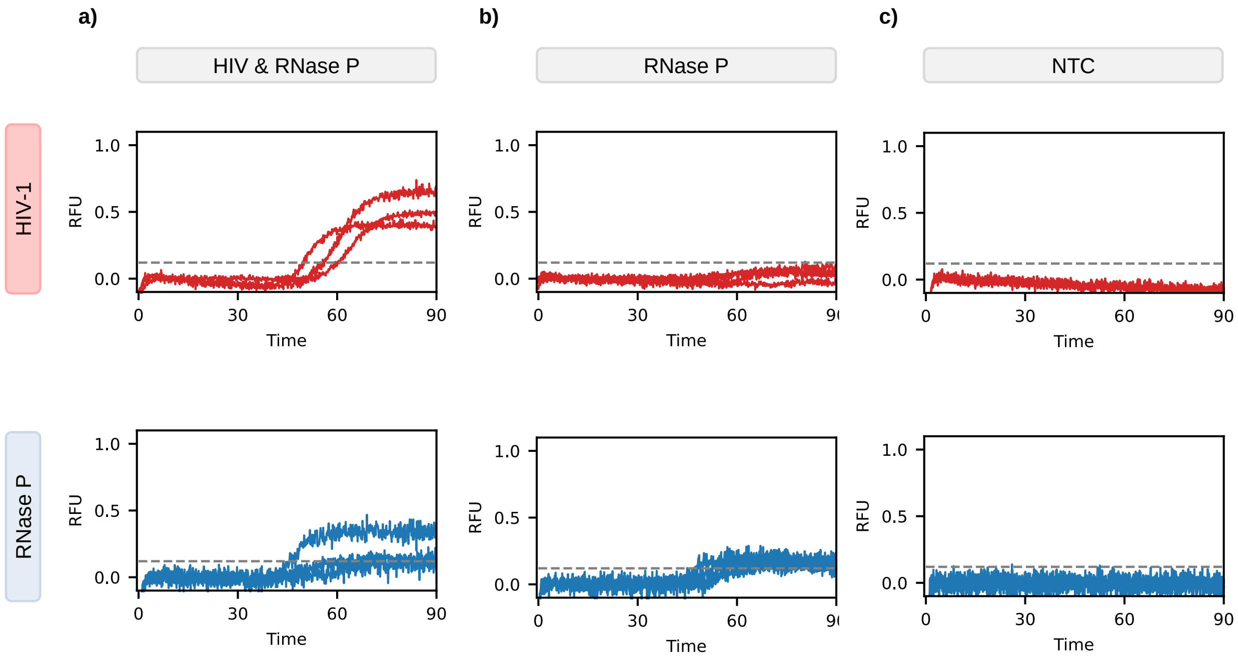

Figure S3. Multiplex cross-reactivity using our portable analyzer. a) The multiplex assay used FAM and HEX fluorophores to distinguish HIV-1 and RNase P, respectively. When both targets are present in the sample, there is exponential amplification for both FAM and HEX. b) For samples with only RNase P, there is only amplification detected in the HEX channel. c) No Target Controls (NTC) show no amplification for either FAM or HEX (HIV-1 or RNase P).

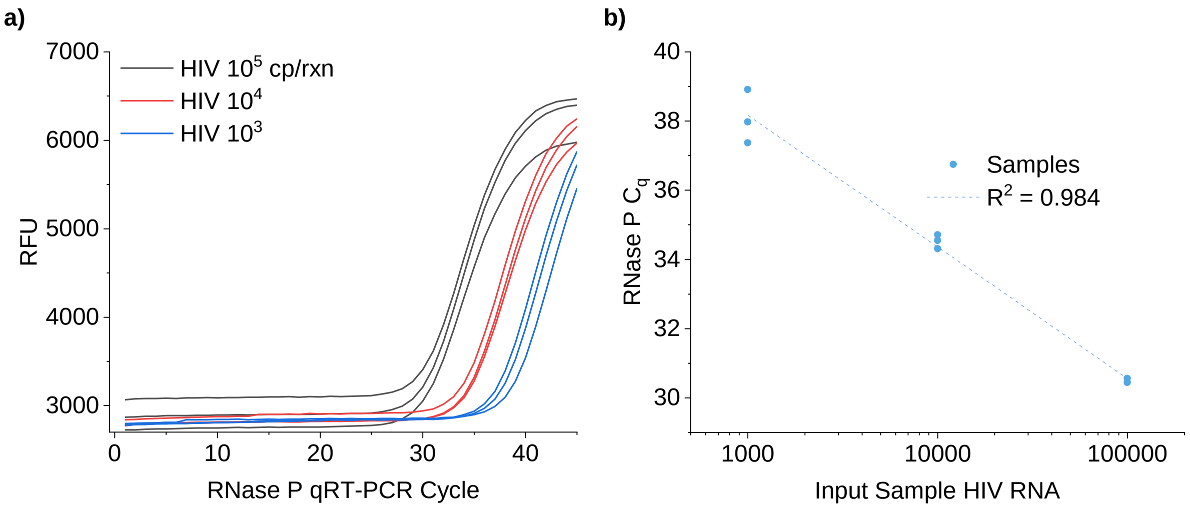

Figure S4. RNase P Validation. a) qRT-PCR amplification curves from HIV Seracare samples at three diluted concentrations, showing RNase P presence. b) PCR Cq showed a linear relationship against input concentration.

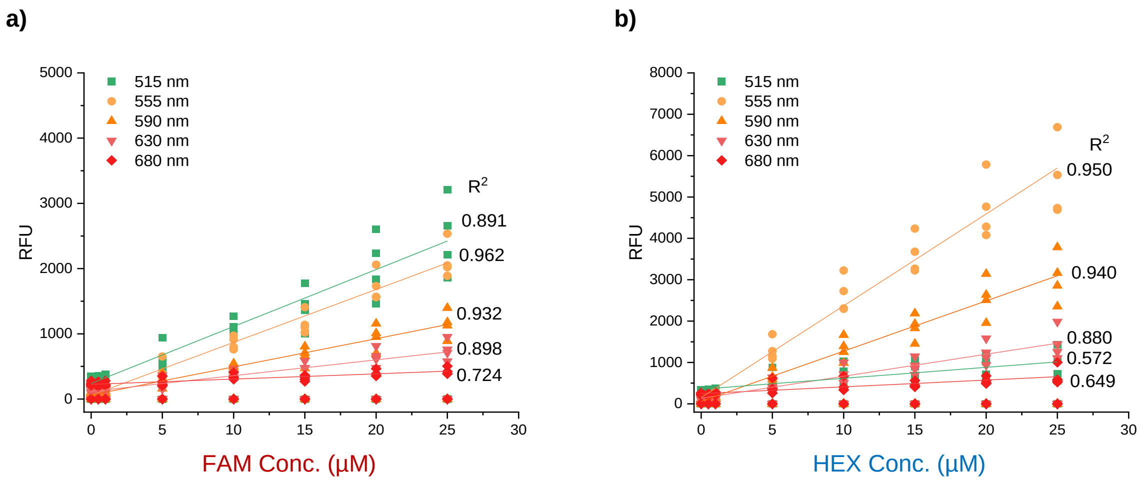

Figure S5. Typical dye performance using single channel detection. a) Color sensor readout from five channels showing the crowded yet accurate detection of increasing FAM dye. b) Identical five channel output but now for increasing concentrations of HEX dye. When used in combination the two dyes are indistinguishable, given that both the 515 nm centered (FAM best) and the 555 nm centered (HEX best) channels are sensitive to the other dye.

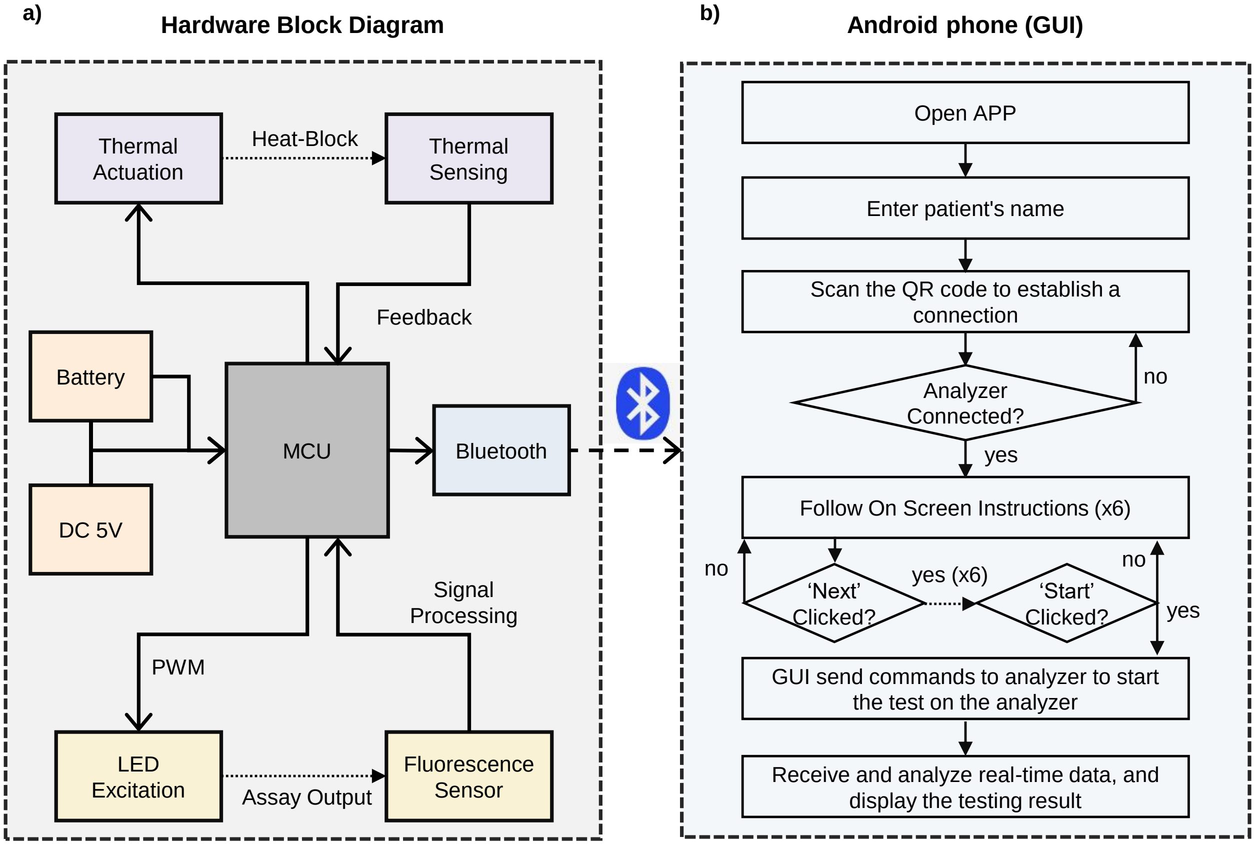

Figure S6. Software and smartphone app. a) Block diagram illustrating the interconnecting components of the portable analyzer. Output includes a resistive heater, an LED for excitation, and a status LED bar. Inputs include temperature feedback from the thermistor and an a wide-spectra color sensor. An Arduino Nano controls all modules and interfaces with an Android phone through Bluetooth. b) Block diagram demonstrating the workflow of the Android smartphone app. The GUI will walk patients through the entire testing workflow, confirming each step has been completed, and will display results on the last page with an option to upload to cloud storage sites.

Supplementary Tables

Table S1. Clinically archived sample performance

Sample Number	1	2	3	4	5	6	7	8	9	10	11	12	13	14	15	16	17	18	19	20	21	22	23
RT PCR HIV	28	27	27	33	34	34	0	0	0	0	0	0	35	35	35	33	32	33	35	35	36	33	33
RT PCR HIV	+	+	+	+	+	+	-	-	-	-	-	-	+	+	+	+	+	+	+	+	+	+	+
RT LAMP HIV	42	41	40	0	72	64	0	0	0	0	0	0	43	42	43	51	56	61	0	0	0	0	0
RT LAMP HIV	+	+	+	-	+	+	-	-	-	-	-	-	+	+	+	+	+	+	-	-	-	-	-
RT LAMP RP	47	45	43	65	75	44	0	53	0	63	0	72	51	48	51	61	61	57	0	0	0	0	0
RT LAMP RP	+	+	+	+	+	+	-	+	-	+	-	+	+	+	+	+	+	+	-	-	-	-	-

Sample Number	24	25	26	27	28	29	30	31	32	33	34	35	36	37	38	39	40	41	42	43	44	45
RT PCR HIV	34	38	30	35	0	0	0	0	0	0	0	0	0	0	0	0	0	0	0	0	0	0
RT PCR HIV	+	+	+	+	-	-	-	-	-	-	-	-	-	-	-	-	-	-	-	-	-	-
RT LAMP HIV	0	63	67	72	0	0	0	0	0	0	0	0	0	0	0	0	0	0	0	0	0	0
RT LAMP HIV	-	+	+	+	-	-	-	-	-	-	-	-	-	-	-	-	-	-	-	-	-	-
RT LAMP RP	0	62	59	49	0	0	0	58	51	55	70	45	49	0	0	0	65	50	60	0	55	0
RT LAMP RP	-	+	+	+	-	-	-	+	+	+	+	+	+	-	-	-	+	+	+	-	+	-

Table S2. Multiplex LAMP assay

Component	Volume (µL)/rxn	Final Concentration
NEB Isothermal Buffer	5.00	1 X
HIV-F3	0.38	0.15 µM
HIV-B3	0.38	0.15 µM
HIV-FIP	0.80	0.8 µM
HIV-BIP	1.20	1.2 µM
HIV-LB	0.60	0.6 µM
HIV-LF	0.60	0.6 µM
HIV-QFIP:Fd	0.40	0.4 µM
RNase P-F3	0.25	0.1 µM
RNase P-B3	0.25	0.1 µM
RNase P-FIP	0.80	0.8 µM
RNase P-BIP	0.80	0.8 µM
RNase P-LFP:Q	0.40	0.4 µM
RNase P-LB	0.40	0.4 µM
Betaine	4.00	0.4 M
MgSO4	3.00	6 mM
dNTP Mix	7.00	1.4 mM
Bst2.0 polymerase	3.44	0.55 U/µl
Warmstart Reverse Transcriptase	1.10	0.33 U/µl
RNA	10.00	10 µl/rxn
UltraPure Water	9.21	-

Table S3. Probe-based RT-LAMP primers

Target	Primer	Sequence (5' -> 3')	GenBank Target
RNase P	F3	TTGATGAGCTGGAGCCA	U94316.1
RNase P	B3	CACCCTCAATGCAGAGTC	U94316.1
RNase P	FIP	GTGTGACCCTGAAGACTCGGTTTTAGCCACTGACTCGGATC	U94316.1
RNase P	BIP	CCTCCGTGATATGGCTCTTCGTTTTTTTCTTACATGGCTCTGGTC	U94316.1
RNase P	LFP	/5HEX/ATGTGGATGGCTGAGTTGTT	U94316.1
RNase P	LB	CATGCTGAGTACTGGACCTC	U94316.1
RNase P	Q	CAGCCATCCACAT/3BHQ_1/	U94316.1
HIV-1	F3	AGTTCCCTTAGATAAAGACTT	AF033819.3
HIV-1	B3	CCTACATACAAATCATCCATGT	AF033819.3
HIV-1	FIP	GTGGAAGCACATTGTACTGATATCTTTTTGGAAGTATACTGCATTTACCAT	AF033819.3
HIV-1	BIP	GGAAAGGATCACCAGCAATATTCCTCTGGATTTTGTTTTCTAAAAGGC	AF033819.3
HIV-1	LB	GCATGACAAAAATCTTAGA	AF033819.3
HIV-1	LF	GGTGTCTCATTGTTTATACTA	AF033819.3
HIV-1	QFIP	/5IABkFQ/GTGGAAGCACATTGTACTGATATCTTTTTGGAAGTATACTGCATTTACCAT	AF033819.3
HIV-1	Fd	GATATCAGTACAATGTGCTTCCAC /36-FAM/	AF033819.3

Table S4. qRT-PCR primers

Target	Primer	Sequence (5' - 3')
HIV-1 pol	Forward	CATGTTTTCAGCATTATCAGAAGGA
HIV-1 pol	Reverse	TGCTTGATGTCCCCCCACT
HIV-1 pol	Probe	FAM-CCACCCCACAAGATTTAAACACCATGCTAA-BHQ1

Table S5. Portable Analyzer Bill of Materials

System	Description	Part#	Function	Unit Cost ($)	Unit Qty.	Ext. Cost ($)
Electronics	Arduino Nano	7630049200173	Microcontroller	6.00	1	6.00
Electronics	Adafruit Bluefruit LE SPI	2633	Bluetooth Low Energy	14.00	1	14.00
Electronics	Capacitor 10uF	P10425CT	Power stabilizing	0.15	1	0.15
Electronics	DC Barrel Power Jack/Connector	PRT-00119	Power Connector	1.25	1	1.25
Electronics	36-pin stripe male header	392	Headpins	4.95	0.1	0.50
Thermal	Aluminum CNC block	CP-0.91-0.91	Heating Stage	5.75	0.25	1.44
Thermal	2Ω Power Resistor	MP725-2.00-FCT	Heater	7.01	1	7.01
Thermal	SMD Resistor, 1kΩ	311-1.00KHRCT	Temperature/EM control	0.01	1	0.01
Thermal	Thermistor	235-1057	Temperature sensing	3.50	1	3.50
Thermal	SMD Resistor, 10kΩ	RNCP0603FTD10K0CT	Temperature control	0.10	1	0.10
Thermal	N Channel Power MOSFET	DMN1019USN-7DICT	Switch for heater and fan	0.35	2	0.70
Optics	Res Cermet Trimmer 500K ohm	3296W-1-501RLF	Adjust LED intensity	0.60	3	1.80
Optics	SMD Resistor, 1kΩ	RNCP0805FTD1K0CT	Optical module	0.03	3	0.08
Optics	SMD Resistor, 10kΩ	RNCP0805FTD10K0CT	Optical module	0.03	8	0.22
Optics	IC Color Sensor	AS7341	Optical detection	3.00	3	9.00
Optics	SMD LED, Blue	1497-1138-1	Fluorescence excitation	0.53	3	1.58
Optics	Capacitor 10uF	P10425CT	Power stabilizing	0.15	3	0.44
Optics	Capacitor 0.1uF	P01425CT	Power stabilizing	0.15	1	0.15
Optics	Voltage Regulator	AP7312-3.3/1.8	Power supplier	0.80	1	0.80
Optics	I2C Multiplexer	TCA9548A	I2C expender for sensor	1.10	1	1.10
Enclosure	ABS Filament (Black)	-	3D platform material	69.00	0.05	3.45
Enclosure	Screws (M2 cap screw)	91290A015	Assembly	0.14	10	1.40
					**Total Cost**	**$63.54**

Supplementary Videos

Supplementary Video S1. (Duration 2m 57s) Demonstration of the testing workflow, using an updated version of RNA extraction.

Supplementary Video S2. (Duration 5m 58s) Initial demonstration of the testing workflow, shown to survey participants prior to the multiple select survey questions.

References

1. Becherer, L. *et al.* Loop-mediated isothermal amplification (LAMP) – review and classification of methods for sequence-specific detection. *Anal. Methods* **12**, 717–746 (2020).
2. A. Nanayakkara, I. & M. White, I. Demonstration of a quantitative triplex LAMP assay with an improved probe-based readout for the detection of MRSA. *Analyst* **144**, 3878–3885 (2019).
3. Ball, C. S. *et al.* Quenching of Unincorporated Amplification Signal Reporters in Reverse-Transcription Loop-Mediated Isothermal Amplification Enabling Bright, Single-Step, Closed-Tube, and Multiplexed Detection of RNA Viruses. *Anal. Chem.* **88**, 3562–3568 (2016).
4. Tanner, N. A., Zhang, Y. & Evans Jr., T. C. Simultaneous Multiple Target Detection in Real-Time Loop-Mediated Isothermal Amplification. *BioTechniques* **53**, 81–89 (2012).
5. Zhang, Y. & Tanner, N. A. Development of Multiplexed Reverse-Transcription Loop-Mediated Isothermal Amplification for Detection of SARS-CoV-2 and Influenza Viral RNA. *BioTechniques* **70**, 167–174 (2021).
6. Bhadra, S. *et al.* Real-Time Sequence-Validated Loop-Mediated Isothermal Amplification Assays for Detection of Middle East Respiratory Syndrome Coronavirus (MERS-CoV). *PLOS ONE* **10**, e0123126 (2015).
7. Jiang, Y. S. *et al.* Robust Strand Exchange Reactions for the Sequence-Specific, Real-Time Detection of Nucleic Acid Amplicons. *Anal. Chem.* **87**, 3314–3320 (2015).
8. Kshirsagar, A., Politza, A. J. & Guan, W. Deep Learning Enabled Universal Multiplexed Fluorescence Detection for Point-of-Care Applications. *ACS Sens.* (2024) doi:10.1021/acssensors.4c00860.
